# CD44 and RHAMM are essential for rapid growth of bladder cancer driven by loss of Glycogen Debranching Enzyme (AGL)

**DOI:** 10.1186/s12885-016-2756-5

**Published:** 2016-09-05

**Authors:** Darby Oldenburg, Yuanbin Ru, Benjamin Weinhaus, Steve Cash, Dan Theodorescu, Sunny Guin

**Affiliations:** 1 Gundersen Medical Foundation, 1300 Badger Street, La Crosse, WI 54601 USA; 2 BioMarin Pharmaceutical Inc, 300 Bel Merin Keys Blvd, Novato, CA 94949 USA; 3 University of Wisconsin-La Crosse, 1725 State St, La Crosse, WI 54601 USA; 4 Department of Surgery (Urology), University of Colorado, 13001 E 17th Pl, Aurora, CO 80045 USA; 5 Department of Pharmacology, University of Colorado, 13001 E 17th Pl, Aurora, CO 80045 USA; 6 University of Colorado Comprehensive Cancer Center, 13001 E 17th Pl, Aurora, CO 80045 USA

**Keywords:** AGL, HAS2, CD44, RHAMM, Bladder cancer

## Abstract

**Background:**

Loss of Amylo-alpha-1-6-glucosidase-4-alpha-glucanotransferase (AGL) drives rapid proliferation of bladder cancer cells by upregulating Hyaluronic acid(HA) Synthase (HAS2) mediated HA synthesis. However the role of HA receptors CD44 and Hyaluronan Mediated Motility Receptor (RHAMM) in regulating the growth of bladder cancer cells driven by loss of AGL has not been studied.

**Methods:**

Western blot analysis and Terminal deoxynucleotidyl transferase (TdT) dUTP Nick-End Labeling (TUNEL) assay was carried out to study cellular apoptosis with HAS2, CD44 and RHAMM loss in bladder cancer cells with and without AGL expression. Proliferation and softagar assays were carried out to study cellular anchorage dependent and independent growth. Clinicopathologic analysis was carried out on bladder cancer patient datasets.

**Results:**

Higher amounts of cleaved Cas3, Cas9 and PARP was observed in AGL low bladder cancer cell with loss of HAS2, CD44 or RHAMM. TUNEL staining showed more apoptotic cells with loss of HAS2, CD44 or RHAMM in AGL low bladder cancer cells. This revealed that bladder cancer cells whose aggressive growth is mediated by loss of AGL are susceptible to apoptosis with loss of HAS2, CD44 or RHAMM. Interestingly loss of either CD44 or RHAMM induces apoptosis in different low AGL expressing bladder cancer cell lines. Growth assays showed that loss of CD44 and RHAMM predominantly inhibit anchorage dependent and independent growth of AGL low bladder cancer cells. Clinicopathologic analysis revealed that high RHAMM mRNA expression is a marker of poor patient outcome in bladder cancer and patients with high RHAMM and low AGL tumor mRNA expression have poor survival.

**Conclusion:**

Our findings strongly point to the importance of the HAS2-HA-CD44/RHAMM pathway for rapid growth of bladder cancer cells with loss of AGL and provides rational for targeting this pathway at various steps for “personalized” treatment of bladder cancer patients based of their AGL expression status.

**Electronic supplementary material:**

The online version of this article (doi:10.1186/s12885-016-2756-5) contains supplementary material, which is available to authorized users.

## Background

Amylo-alpha-1-6-glucosidase-4-alpha-glucanotransferase (AGL) and glycogen phosphorylase (PYG) isoforms are responsible for glycogen breakdown (glycogenolysis) in humans [1]. Inactivation of AGL leads to buildup of abnormal glycogen in the liver, heart and skeletal muscle leading to Glycogen Storage Disease III (GSD III) [2, 3], a condition with good prognosis when treated by high protein and complex carbohydrate diet [2]. We have identified AGL as a tumor growth suppressor and prognostic marker in human bladder cancer, for the first time showing AGL plays a role in cancer biology [4].

We have identified that AGL’s role in tumor biology is independent of its enzymatic activity and is not due to changes in glycogenolysis [4]. We have further identified that loss of AGL promotes rapid cancer cell proliferation dependent on extracellular glucose, Serine Hydroxymethyltransferase 2 (SHMT2) driven glycine synthesis and Hyaluronic Acid (HA) Synthase 2 (HAS2) mediated HA synthesis [4, 5]. Using genetic manipulation and chemical inhibition of HA synthesis we have demonstrated that HAS2 dependent HA synthesis is a major driven of tumor growth with AGL loss [5].

HA is known to interact with many cell surface proteins to activate downstream signaling pathways [6]. CD44 and Hyaluronan Mediated Motility Receptor (RHAMM) are the two major cell surface proteins HA bind to trigger downstream signaling which promotes tumor growth and metastasis [6–8]. Here we aim to study the role of CD44 and RHAMM, downstream of HA, in rapid growth of bladder cancer cells driven by low AGL expression. We identified that loss of either CD44 or RHAMM induce apoptosis in specific low AGL bladder cancer cell lines. CD44 and RHAMM both play a role in inhibiting anchorage dependent and independent growth of bladder cancer cells with low AGL expression. We also identified that RHAMM mRNA expression alone and in combination with AGL mRNA expression serves as a prognostic marker in bladder cancer.

## Methods

### Cell line and biochemical reagents

UMUC3, T24T and MGHU4 control (shCTL) and AGL (shAGL) depleted human bladder cancer cells were, cultured and used as described [4, 5]. AGL knockdown in these were achieved using shRNA TRCN0000035082 (Sigma-Aldrich) as described previously [4, 5]. These three bladder cancer cell lines were chosen for the study because they show an induction in growth with AGL loss, hence serve as good model cell lines to study AGL biology in bladder cancer [4, 5]. 4-Methylumbelliferone (4-MU, cat. # M1508-10G) was obtained from Sigma-Aldrich. HA (cat. # GLR001) was obtained from R&D systems (Minneapolis, MN). siRNA sequences 5’-GGTTTGTGATTCAGACACT-3’ was used at a concentration of 50nM to knockdown HAS2 (siHAS2) as previously reported [5]. siGENOME SMARTpool siRNAs were used to knockdown CD44 (M-009999-03-0005, siCD44) and RHAMM (M-010409-01-0005, siRHAMM) at a concentration of 20nM. siRNA’s were purchased from Dharmacon (Lafayette, CO) and transfected using Lipofectamine RNAiMAX (Invitrogen) using manufacturer instructions. A second siRNA sequence 5’-GCAGATCGATTTGAATATA-3’ for CD44 (siCD44-2) and 5’-GAGCTCAAATCAAGAATAT-3’ for RHAMM (siRHAMM-2), purchased from Dharmacon (Lafayette, CO), were also used at a concentration of 20nM to knockdown CD44 and RHAMM respectively using above mentioned protocol. A non-specific siRNA (siCTL, 5′-CGTACGCGGAATACTTCGA-3′) was used as control for all the experiments. Human bladder cancer cell lines UMUC3, T24T and MGHU4 were authenticated by the University of Colorado PPSR core using an Applied Biosystems Profiler Plus Kit which analyzed 9 STR loci (Life Technologies 4303326). After authentication cells were frozen within 1–2 weeks. Vials of cells were resuscitated less than 2 months prior to being used in experiments in this study.

### PCR and western blot

HAS2 mRNA expression was determined by the ΔΔCT method [4, 5] with GAPDH as control for human bladder cancer cell lines. Expression was normalized to cells transduced with control plasmid (shCTL) transfected with control siRNA (siCTL) to determine HAS2 gene expression and knockdown in control (shCTL) and AGL knockdown (shAGL) cells with HAS2 siRNA treatment. When shAGL cells were transfected with control siRNA or siRNA specific to CD44 or RHAMM, HAS2 gene expression was determined by normalizing to shAGL cells transfected with siCTL. HAS 2 primer: forward 5’- TCCCGGTGAGACAGATGAGT-3’ reverse 5’ GGCTGGGTCAAGCATAGTGT-3’; GAPDH primer: forward 5’-TCTTTTGCGTCGCCAGCCGA 3’ reverse 5’- ACCAGGCGCCCAATACGACC-3’ were used for the RT-PCR experiments.

Antibodies used for westerns were anti-AGL (Agrisera, Vannas, Sweden) and anti- α tubulin (Calbiochem, San Diego, CA), Actin (GeneTex, Irvine, CA), CD44 (Cell Signaling), RHAMM (Abcam, Cambridge, MA), apoptotic antibody sampler kit (Cell Signaling), ERK (Cell Signaling), p-ERK (Cells Signaling), DR5 (Cell Signaling) and Fas (Cell Signaling). HRP (Cell Signaling) labeled mouse or rabbit secondary antibodies were used chemiluminescence using ECL (Pierce, Rockford, IL).

### Terminal deoxynucleotidyl transferase (TdT) dUTP Nick-End Labeling (TUNEL) Assay

shCTL and shAGL bladder cancer cells were plated in chambered slides and treated with control siRNA or siRNA against HAS2, CD44 or RHAMM. 48 h after transfection TUNEL assay was carried out using DeadEnd™ Colorimetric TUNEL System from Promega following manufacturer instruction. Images of cells taken using Olympus IX71 microscope at a magnification of 40X. Images were analyzed and quantified using ImageJ.

### Anchorage dependent and independent proliferation

Anchorage dependent and independent proliferation was measured as before [4, 9]. Briefly, shCTL and shAGL bladder cancer cells were transfected with control siRNA or siRNA targeting CD44 or RHAMM. 72 h after transfection anchorage-independent growth was assessed by plating cells in 0.4 % agar in triplicate. Colonies were stained with Nitro-BT (Sigma) and counted using Image J. Cell proliferation and viability was assessed by plating 10^3^ cells per well in 96-well plates in triplicate for proliferation studies. CyQUANT® Cell Proliferation Assay (Thermo Fisher Scientific) was carried out according to manufacturer instruction to measure cell proliferation.

### HA ELISA

Fresh media is applied 48 h after CD44 or RHAMM siRNA transfection in shAGL cells followed by HA analysis by ELISA 24 h later. HA ELISA was conducted as per manufacturer instructions using TECO® HA ELISA kit.

### Immunofluorescence

UMUC3 and T24T cells with and without AGL expression were plated in chambered slides. 24 h later cells were washed with PBS, fixed with 4 % paraformaldehyde, permealized with 0.2 % Triton X-100, blocked with 1 % bovine serum albumin (BSA) followed by treatment with anti-RHAMM antibody (Cat no. 185728, Abcam, Cambridge, MA). Secondary antibody used was tagged with Alexa Fluor 488 Nfrom Thermo Fisher Scientific. Slides were mounted with ProLong^(R)^ Diamond Antifade Mountant with DAPI (Thermo Fisher Scientific). Images of cells taken using Olympus IX71 microscope at a magnification of 40X.

### Patient and statistical analysis

Patient microarray and clinicopathologic information of patient datasets [10, 11] is shown in Additional file 1: Table S1. Raw microarray data were processed and normalized by the Robust Multi-array Average algorithm implemented in the *affy* package in R [12]. In case of multiple probe sets for one gene, the probe with the highest mean expression across all samples was selected to represent the gene’s expression. Gene expression differences between two groups of samples (tumor vs. normal, high grade vs. low grade, and muscle invasive (MI) vs. non-muscle invasive tumors (NMI)) were tested by Wilcoxon rank sum tests. Associations of categorical gene expression with survival were examined by Cox proportional hazards models and logrank tests. Data from in vitro experiments were analyzed by 2-tailed Student *t*-test with unequal variances. Error bars denote standard deviation of the mean as indicated. "n" in the Figure Legends represents the number of replicates for a particular sample.

## Results

### Inhibition of HA synthesis induce apoptosis in low AGL bladder cancer cells

**Fig. 1:**
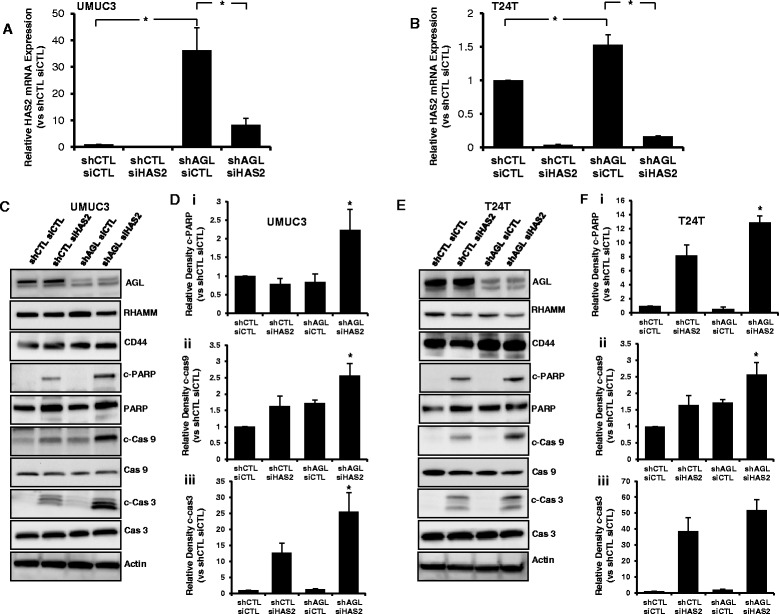
HAS2 loss and apoptosis in bladder cancer cells +/− AGL. **a**, **b** qRT-PCR demonstrating efficacy of HAS2 depletion in UMUC3 and T24T control (shCTL) and AGL knockdown (shAGL) cells. Cells were plated and 24 h later transfected with scrambled (siCTL) or directed siRNA against HAS2 (siHAS2). Details of siRNA used are in Materials and Methods. Cells were harvested at 48 h for mRNA followed by qRT-PCR analysis (*n* = 3). **c** 48 h after UMUC3 shCTL and shAGL cells were transfected with scrambled siRNA (siCTL) or siRNA against HAS2 (siHAS2), cells were lysed and expression of CD44, RHAMM and the proteins involved in the apoptotic pathway were detected by Western blot. **d** Densitometric analysis of cleaved apoptotic proteins normalized to total protein and the UMUC3 shCTL siCTL sample (*n* = 3). **e** 48 h after T24T shCTL and shAGL were transfected with scrambled siRNA (siCTL) or siRNA against HAS2 (siHAS2), cells were lysed and expression of CD44, RHAMM and the proteins involved in the apoptotic pathway were detected by Western blot. **f** Densitometric analysis of cleaved apoptotic proteins normalized to total protein and the T24T shCTL siCTL sample (*n* = 3). Results are shown as mean ± SD, *P < 0.05

HA is known to induce tumor growth and metastasis by interacting with its two main receptors CD44 and RHAMM [7, 8]. Earlier studies have shown that inhibition of HA synthesis results in reduction of CD44 and RHAMM expression, and also leads to cellular apoptosis [13, 14]. We have earlier shown that loss of AGL induces rapid growth of bladder cancer cells via HAS2 mediated HA synthesis [5]. Here we study the role of HA’s two main receptors in bladder cancer growth driven by low AGL expression. Bladder cancer cells UMUC3 and T24T cells with (shCTL) and without (shAGL) AGL was used in this study. These bladder cancer cells transduced with control shRNA (shCTL) and shRNA against AGL (shAGL) has been used by us in previous research projects [4, 5]. We have previously demonstrated knockdown of HAS2 reduces HA synthesis predominantly in shAGL cells [5]. Here HAS2 was knocked down in UMUC3 and T24T shCTL and shAGL cells using the same siRNA against HAS2 (siHAS2) used previously [5]. We observed higher HAS2 expression in UMUC3 and T24T shAGL cells (Fig. 1a, b) as reported previously and the siHAS2 reduces its expression in the shCTL and shAGL bladder cancer cells (Fig. 1a, b).

Interestingly loss of HAS2 does not reduce expression of CD44 and RHAMM in bladder cancer cells irrespective of AGL expression status (Fig. 1c, e). 4MU, a well-known inhibitor of HA synthesis, has been shown by us to inhibit HA synthesis and growth of bladder cancer cells driven by loss of AGL [5]. 4MU, like HAS2, has been shown to reduce CD44 and RHAMM receptor expression in cancer cells [13]. We treated UMUC3 and T24T shCTL and shAGL cells with 500 μM 4MU, a concentration at which it reduces HA synthesis. However 4MU treatment did not have a major impact on the expression of CD44 and RHAMM in UMUC3 or T24T shCTL and shAGL cells (Additional file 1: Figure S1). Next we added low molecular weight hyaluronic acid (LMW HA, 15–40 kDa) at a concentration of 50 and 100 μg/ml on the above mentioned cells. LMW HA at 100 μg/ml partially rescued the growth inhibition caused by 4MU treatment of AGL knockdown bladder cancer cells [5]. Treatment with superfluous amounts of LMW HA had little impact on CD44 and RHAMM expression of UMUC3 and T24T bladder cancer cells irrespective of AGL expression status (Additional file 1: Figure S2). These experiments show that loss of HA synthesis by genetic alteration (inhibition of HAS2 expression) or by an inhibitor (4MU) or addition of superfluous HA have little effect on CD44 and RHAMM expression in UMUC3 and T24T bladder cancer cells.

Next we looked into cellular apoptotic pathway with loss of HAS2 in bladder cancer cells with and without AGL. SiHAS2 predominantly induced apoptosis of UMUC3 shAGL cells as shown by higher levels of cleaved PARP, Cas9 and Cas3 (Fig. 1c, d). In T24T cells loss of HAS2 induced apoptosis in both shCTL and shAGL cells but induction of apoptosis was higher in shAGL cells as observed by higher levels of cleaved PARP, Cas9 and Cas3 (Fig. 1e, f). These experiments suggest that AGL low rapid growing bladder cancer cells are vulnerable to apoptosis on inhibition of HA synthesis.

### Loss of either CD44 or RHAMM Induce apoptosis in low AGL expressing bladder cancer cells

**Fig. 2:**
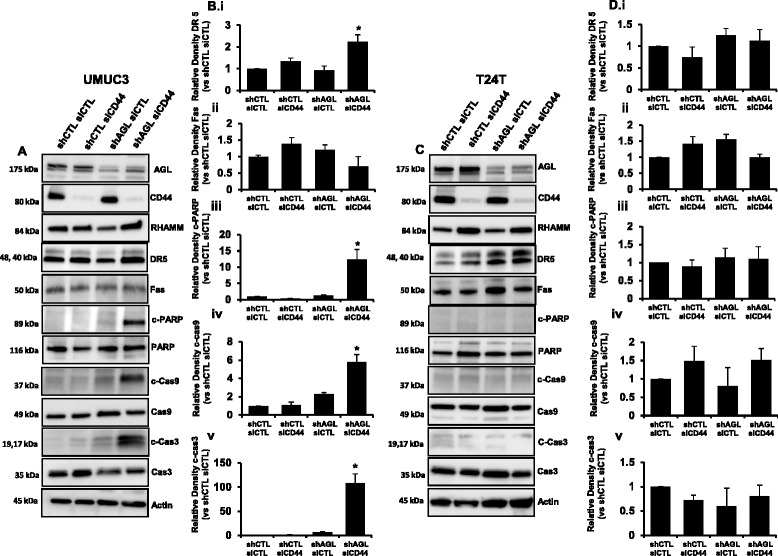
CD44 loss and apoptosis in bladder cancer cells +/− AGL. **a** UMUC3 shCTL and shAGL cells were plated and 24 h later transfected with scrambled siRNA (siCTL) or siGENOME SMARTpool siRNA against CD44(siCD44). Details of siRNA are in Material and Methods. Cells were lysed 48 h after transfection and Western blot was carried out for proteins involved in apoptosis. **b** Densitometric analysis of cleaved apoptotic proteins normalized to total protein and the UMUC3 shCTL siCTL sample (*n* = 3). DR5 and Fas normalized to Actin and the UMUC3 shCTL siCTL sample (*n* = 3). Results are shown as mean ± SD, *P < 0.05. **c** T24T shCTL and shAGL cells were plated and 24 h later transfected with scrambled siRNA (siCTL) or siGENOME SMARTpool siRNA against CD44 (siCD44). Details of siRNA are in Material and Methods. Cells were lysed 48 h after transfection and Western blot was carried out for proteins involved in apoptosis. **d** Densitometric analysis of cleaved apoptotic proteins normalized to total protein and the T24T shCTL siCTL sample (*n* = 3). DR5 and Fas normalized to Actin and the shCTL siCTL sample (*n* = 3). Results are shown as mean ± SD, *P < 0.05

**Fig. 3:**
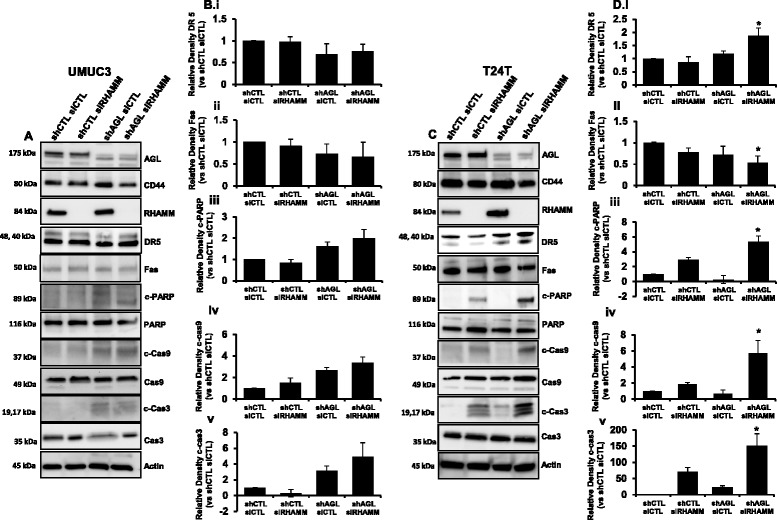
RHAMM loss and apoptosis in bladder cancer cells +/− AGL. **a** UMUC3 shCTL and shAGL cells were plated and 24 h later transfected with scrambled siRNA (siCTL) or siGENOME SMARTpool siRNA against RHAMM (siRHAMM). Details of siRNA are in Material and Methods. Cells were lysed 48 h after transfection and Western blot was carried out for proteins involved in apoptosis. **b** Densitometric analysis of cleaved apoptotic proteins normalized to total protein and the UMUC3 shCTL siCTL sample (*n* = 3). DR5 and Fas normalized to Actin and the UMUC3 shCTL siCTL sample (*n* = 3). Results are shown as mean ± SD, *P < 0.05. **c** T24T shCTL and shAGL cells were plated and 24 h later transfected with scrambled siRNA (siCTL) or siGENOME SMARTpool siRNA against RHAMM (siRHAMM). Details of siRNA are in Material and Methods. Cells were lysed 48 h after transfection and Western blot was carried out for proteins involved in apoptosis. **d** Densitometric analysis of cleaved apoptotic proteins normalized to total protein and the T24T shCTL siCTL sample (*n* = 3). DR5 and Fas normalized to Actin and the UMUC3 shCTL siCTL sample (*n* = 3). Results are shown as mean ± SD, *P < 0.05

It has been shown that inhibition of HA signaling induce apoptosis by activating Death receptor signaling [13, 15]. Since loss of HAS2 induced apoptosis predominantly in the rapid growing shAGL bladder cancer cells we decided to investigate whether loss of the two dominant HA receptor CD44 and RHAMM, result in similar apoptotic induction of shAGL bladder cancer cells. We knocked down CD44 and RHAMM individually in UMUC3 and T24T cells with and without AGL to study their role in apoptosis. Knockdown of CD44 using siGENOME SMARTpool siRNA (siCD44) was able to induces Death Receptor 5 (DR5) expression followed by apoptotic signaling in UMUC3 shAGL cells as seen by increased levels of cleaved PARP, Cas9 and Cas3 (Fig. 2a, b). Fas has also been shown as an inducer of apoptosis with inhibition of HA signaling [13, 15], however we did not see any increase in Fas expression with CD44 loss (Fig. 2a, b). Interestingly loss of CD44 did not induce DR5 or apoptotic signaling in T24T bladder cancer cells with or without AGL expression. The experiments were repeated using a second siRNA (siCD44-2) against CD44. SiCD44-2 also induced apoptosis only in UMUC3 shAGL cells (Additional file 1: Figure S3). Similarly genetic knockdown of RHAMM using siGENOME SMARTpool siRNA (siRHAMM) was carried out in UMUC3, T24T shCTL and shAGL cells. Surprisingly knockdown of RHAMM induced DR5 expression and apoptotic signaling predominantly in T24T shAGL cells and not in UMUC3 shCTL or shAGL cells (Fig. 3). A second siRNA against RHAMM (siRHAMM-2) yielded similar results (Additional file 1: Figure S4).

Since loss of either CD44 or RHAMM induced apoptosis in the two different shAGL bladder cancer cells (UMUC3 and T24T respectively) used, we decided to include a third cell line into our study. MGHU4 bladder cancer cells, with loss of AGL, have shown rapid growth driven by the HAS2/HA axis [5]. Knockdown of RHAMM and not CD44 induced apoptosis in MGHU4 shAGL cells as seen by increased levels of c-Cas3 (Additional file 1: Figure S5). Thus showing that loss of RHAMM is a major inducer of apoptotic signaling in bladder cancer cells driven by AGL loss.

RHAMM is known to have different function based of its cellular localization [16, 17]. HA is known to regulate RHAMM function irrespective of its cellular localization [16–19]. Immunofluorescence analysis showed that in UMUC3 shCTL cells RHAMM expression is predominantly cytoplasmic (Additional file 1: Figure S6A). However UMUC3 shAGL cells have RHAMM staining pattern which suggest it is localizes with microtubules and fluorescent punctas in the nuclear region suggesting its dominant presence in the centrosomes (Additional file 1: Figure S6A) [18, 20, 21]. This shows in UMUC3 cells, there is a major change in RHAMM localization with AGL loss. A previous study have shown that HA treatment can result in RHAMM localization to nucleus and centrosomes [18]. We speculate that increase in HA synthesis with AGL loss is driving RHAMM to localize with microtubules and centrosomes in UMUC3 cells, where it is involved in cell division and do not induce apoptosis when the protein is genetically inhibited. In T24T cells irrespective of AGL expression status, RHAMM is present predominantly in the cytoplasm and also in the centrosomes as suggested by the fluorescent punctas in the nucleus of some cells (Additional file 1: Figure S6B). We think loss of RHAMM results in apoptosis of AGL knockdown cells when it is present in the cytoplasm as seen in T24T shAGL cells.

Since RHAMM is known to activate ERK by directly interacting with it or by activating MAPK signaling pathway by interacting with cell surface receptors [16, 17] we looked into activated ERK in UMUC3 and T24T shCTL and shAGL cells. There was no significant difference in ERK activation in these cells with and without AGL (Additional file 1: Figure S6C, D). Loss of RHAMM reduced ERK activation in both UMUC3 and T24T shCTL and shAGL cells (Additional file 1: Figure S6C, D), suggesting cellular localization of RHAMM did not impact ERK activation and downstream signaling driven by ERK in these bladder cancer cells.

We further looked into HAS2 expression and HA synthesis in UMUC3 and T24T shAGL cells with CD44 and RHAMM loss. In UMUC3 shAGL cells loss of CD44 induced apoptosis (Fig. 2a, b) and also majorly inhibited HAS2 expression and HA synthesis (Additional file 1: Figure S7A, C) where as in T24T shAGL cells loss of RHAMM induced apoptosis (Fig. 3c, d) and was responsible for greater reduction of HAS2 expression and HA synthesis (Additional file 1: Figure S7B, D). These outcomes illustrate that specific AGL knockdown bladder cancer cell lines undergo apoptosis with inhibition of either CD44 or RHAMM and loss of this particular HA receptor also inhibit HA synthesis by a feedback mechanism. This also provides evidence that CD44 and RHAMM are involved in HAS2/HA signaling in bladder cancer cells with loss of AGL.

**Fig. 4:**
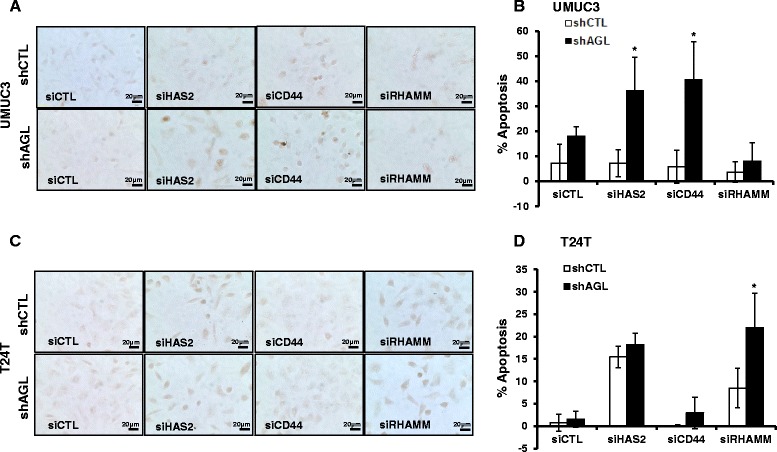
TUNEL assay to detect apoptosis with HAS2, CD44 or RHAMM loss in bladder cancer cells +/− AGL. **a**, **b** UMUC3 and T24T shCTL and shAGL cells were plated in chambered slides and 24 h later transfected with scrambled siRNA or siRNA against HAS2 (siHAS2), CD44 (siCD44) or RHAMM (siRHAMM). Details of siRNA are in Material and Methods. 48 h after transfection cells were subjected to TUNEL assay according to manufacturer protocol as mentioned in Material and Methods. Images were taken at a 40X magnification using Olympus IX71 microscope. **c**, **d** Quantification of TUNEL staining in UMUC3 and T24T shCTL and shAGL cells with the different gene knockdowns (*n* = 3). TUNEL staining quantified using ImageJ. Results are shown as mean ± SD, *P < 0.05

After observing an increase in cleaved caspases with loss of HAS2, CD44 or RHAMM in UMUC3 and T24T shAGL cells, we carried out TUNEL assay to detect the % of cells undergoing apoptosis. Since an increase in cleaved caspases can also be a result of a few cells undergoing apoptosis with a stronger biochemical apoptotic signaling. UMUC3, T24T shCTL and shAGL cells were subjected to TUNEL staining 48 h after transfection with siHAS2, siCD44 or siRHAMM. Quantification of the TUNEL stain showed that 35 to 40 % (*P* < 0.05) UMUC3 shAGL cells undergoes apoptosis with loss of HAS2 and CD44. RHAMM knockdown has no impact on apoptosis of UMUC3 shAGL cells (Fig. 4a, b). These results are consistent with previously observed apoptotic signaling which showed loss of RHAMM did not induce death receptor and apoptotic signaling in UMUC3 shAGL cells (Figs. 2 and 3). Less than 10 % UMUC3 shCTL cells underwent apoptosis with loss of HAS2, CD44 or RHAMM (Fig. 4b). These observations along with our previous work show that parental UMUC3 cells are not vulnerable to inhibition of HAS2/HA-CD44-RHAMM signaling, whereas loss of AGL makes these cells depend on HA signaling.

Our experiments with T24T bladder cancer cells show that 15 to 18 % of T24T shCTL and shAGL cells undergo apoptosis with loss of HAS2 (Fig. 4c, d). Biochemical apoptotic signaling showed that T24T shAGL cells had higher levels of cleaved PARP, Cas9 and Cas3 compared to T24T shCTL cells with HAS2 knockdown (Fig. 1). This suggests that T24T shAGL cells are undergoing a stronger biochemical apoptotic signaling indicated by higher levels of cleaved proteins but the actual number of cells undergoing apoptosis is the same as T24T shCTL cells. Loss of RHAMM induced significantly higher (*P* < 0.05) apoptosis in T24T shAGL cells compared to shCTL cells (Fig. 4c, d) which is consistent with the previously observed apoptotic signaling which showed loss of RHAMM induced death receptor and apoptotic cell signal predominantly in T24T shAGL cells (Figs. 2 and 3). CD44 loss did not impact apoptosis of T24T shCTL or shAGL cells (Fig. 4c, d), since death receptor and apoptotic signaling was not induced with CD44 knockdown in T24T cells +/− AGL expression (Figs. 2 and 3).

### Loss of CD44 and rhamm inhibit growth of bladder cancer cells driven by loss of AGL

**Fig. 5:**
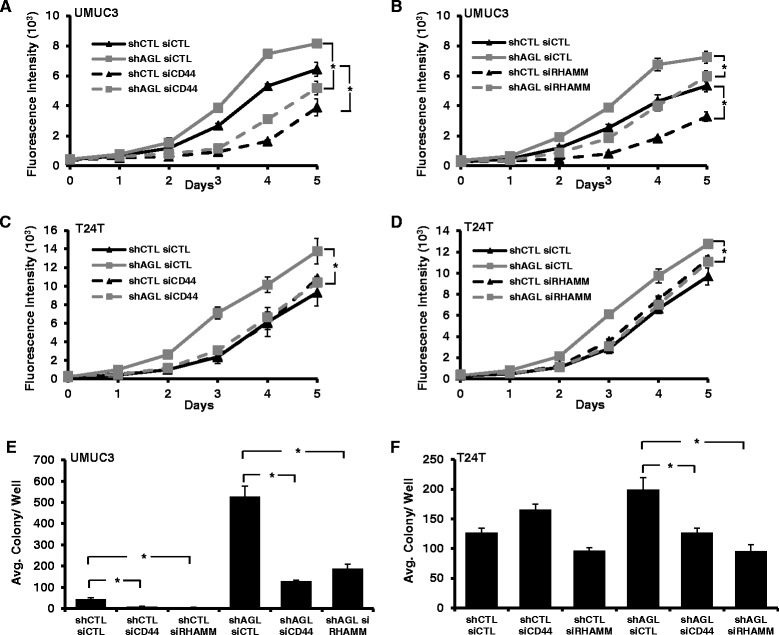
Anchorage dependent and independent growth with CD44 or RHAMM loss in bladder cancer cells +/− AGL. **a**–**d** 72 h after UMUC3 or T24T shCTL and shAGL were transfected with siCTL, siCD44 or siRHAMM, they were plated for monolayer growth (*n* = 6) in 96-welled plate (10^3^ cells/well) for 5 days followed by CyQUANT assay. **e**, **f** 72 h after UMUC3 or T24T shCTL and shAGL were transfected with siRNA against CD44 (siCD44) or RHAMM (siRHAMM) they were plated in agar for evaluation of anchorage independent growth (15x10^3^ cells/well) in 6 well plate (*n* = 3). Results are shown as mean ± SD, *P < 0.05

Loss of AGL promotes anchorage dependent and independent growth of bladder cancer cells [4]. Here we explore the role of CD44 and RHAMM in the rapid growth of bladder cancer cells mediated by AGL loss. UMUC3, T24T shCTL and shAGL cells were plated for anchorage dependent and independent growth assay 72 h after knockdown of CD44 or RHAMM using siRNAs. The cells were trypsinized 72 h after the siRNA transfections, counted and replated for the anchorage dependent and independent growth assays. Thus we got rid of the cells which have undergone apoptosis in the growth assay, since the maximum apoptosis was observed 48 h after gene knockdown. In UMUC3 cells loss of CD44 and RHAMM reduced the anchorage dependent proliferation of shCTL and shAGL cells (Fig. 5a, b) where as in T24T cells loss of CD44 and RHAMM only reduced the proliferation of shAGL cells (Fig. 5c, d). We also introduced MGHU4 cells in our study. Loss of CD44 and RHAMM reduces the anchorage dependent proliferation of MGHU4 shAGL cells (Additional file 1: Figure S8). Similarly in anchorage independent growth assay loss of CD44 or RHAMM reduced the growth of both UMUC3 shCTL and shAGL cells (Fig. 5e) however only reduced the growth of T24T shAGL cells (Fig. 5f). CD44 and RHAMM regulate numerous downstream proliferative signaling pathways [18, 22, 23]. These experiments suggest that the proliferative pathways driven by CD44 and RHAMM are important for anchorage independent and dependent growth of some bladder cancer cells (UMUC3) irrespective of AGL expression status but in others (T24T and MGHU4), there proliferative pathways are more important for the fast growing AGL knockdown cells. These experiments imply that increase in HA synthesis with AGL loss results in increased CD44 and RHAMM dependent proliferative signaling in T24T and MGHU4 shAGL cells.

### The relevance of AGL, CD44 and RHAMM in human bladder cancer

**Fig. 6:**
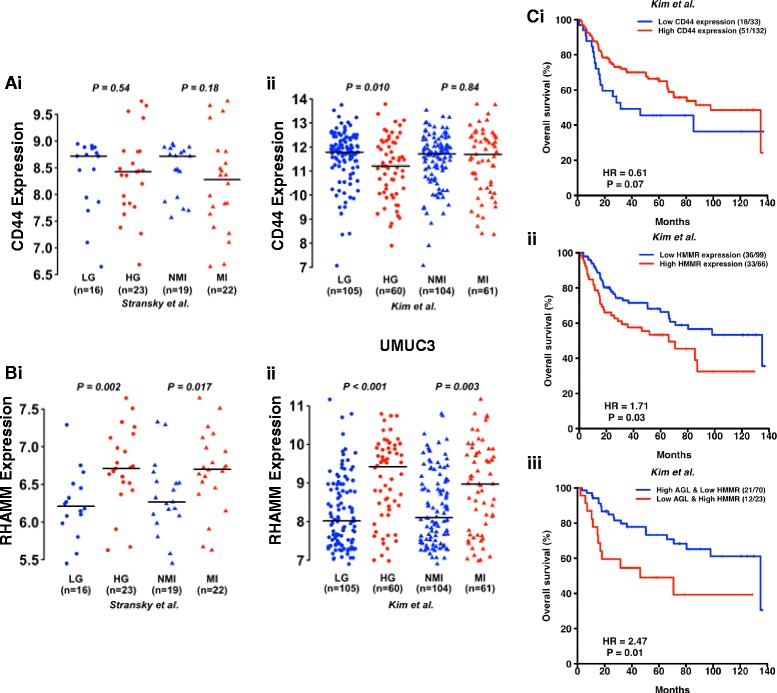
Relationship of CD44, RHAMM and AGL mRNA to clinicopathologic variables in human bladder cancer. **a**, **b** CD44 and RHAMM mRNA expression in high grade (HG) and muscle invasive (MI) bladder tumors compared to low grade (LG) and non-muscle invasive (NMI) bladder tumors in two independent patient datasets (*i*) Stransky et al. [11] and (*ii*) Kim et al. [10] (Additional file 1: Table S1). **c** Kaplan Meier analysis of categorized (high/low) mRNA levels of *i)* CD44; *ii)* RHAMM and *iii)* AGL and RHAMM and overall survival in the Kim et al. [10] bladder patient dataset. Hazard Ratios (HR) and logrank *P* values are shown. High- and low-expression groups were determined by an optimal cutoff that gave the best *p*-value and was selected from nine different percentiles (from 10th to 90th). The optimal cutoff was 20th percentile for CD44, 60th percentile for RHAMM and 30th percentile for AGL

We investigated into RHAMM, CD44 mRNA as a prognostic marker in bladder cancer. CD44 mRNA is not differentially expressed between muscle and non-muscle invasive tumors in two independent patient datasets [10, 11] (Fig. 6a). However mRNA expression of CD44 is low in high grade tumors with *P* = 0.01 in one of the two independent datasets studied (Fig. 6a). High RHAMM mRNA expression was observed in high grade and muscle invasive disease in two independent patient datasets with significant *P* value (Fig. 6b). Analysis of patient overall survival shows that low CD44 expression tends to poor overall survival of bladder cancer patients in Kim et al. [10] dataset (Fig. 6c*i*), however the data is not statistically significant (*HR* = 0.61, *P* = 0.07). A similar analysis showed high mRNA expression of RHAMM predicts poor overall survival for bladder cancer patients (*HR* = 1.71, *P* = 0.03; Fig. 6c*ii*) in Kim et al. [10] dataset. These experiments suggest that high CD44 mRNA expression is not a predictor of poor bladder cancer patient outcome even though CD44 is an important contributor in tumor growth and metastasis.

Since high RHAMM expression is a predictor of poor outcome in bladder cancer patients, we examined the utility of combining AGL and RHAMM expression in stratifying bladder patient outcome. The primary objective here was to determine if such expression levels could eventually be used to identify the optimal patient cohort who may be enrolled in future clinical trials with inhibitors of RHAMM or HA signaling. The secondary objective was to lead credence to the hypothesis that AGL affects tumor biology by RHAMM downstream of HAS2/HA axis as well as other effectors such as SHMT2 [4]. Kaplan-Meier survival using the same cutoffs as for individual RHAMM (Fig. 6c*ii*) and AGL also revealed a significant stratification of survival but with a somewhat better HR of 2.47 (Fig. 6c*iii*). Importantly, this analysis indicated that combining these two variables enhanced the magnitude of the stratification as measured by the HR compared to using either variable alone.

## Discussion

We have previously established that loss of AGL drives aggressive bladder cancer growth via HAS2 mediated HA synthesis and provided preclinical evidence that targeting the HAS2/HA axis is possibly therapeutically beneficial for bladder cancer patients with low AGL expression [5]. HA interacts with numerous cell surface proteins [6]. Of these, CD44 and RHAMM have been extensively studied in cancer biology [7]. It is well known that downstream signaling prompted by CD44 and RHAMM on interaction with HA is crucial for HA mediated tumor growth and metastasis in various tumor types including bladder [24–27].

Here we focus on the importance of CD44 and RHAMM in driving the rapid growth of bladder cancer cells with low AGL expression. The aim of the study was to identify if aggressive bladder tumor growth mediated by AGL loss depends on either CD44 or RHAMM or both. This would point to new therapeutic avenue for bladder cancer patients based of their AGL expression levels. We used established bladder cancer cell lines in our experiments to test our hypothesis followed by analysis of bladder cancer patient samples to emphasize the clinical relevance of our work. Our study resulted in some interesting findings which merits discussion.

Previous studies have reported that genetic or chemical inhibition of HA synthesis by knockdown of the HAS enzymes or treatment with 4MU results in decrease of CD44 and RHAMM protein expression [6, 7, 28] which has been used as a readout of HA based cell signaling inhibition. However it is not known how inhibition of HA synthesis regulate CD44 and RHAMM expression. Interestingly when we knocked down HAS2 or treated with 4MU, UMUC3 and T24T bladder cancer cells +/− AGL expression had no change in their CD44 and RHAMM expression. Addition of superfluous amounts of HA to these cells did not impact CD44 and RHAMM expression. However knockdown of CD44 and RHAMM did reduce HAS2 expression and HA synthesis in UMUC3 and T24T shAGL cells providing evidence that there HA receptors are involved in HAS2/HA signaling in the rapid growing shAGL cells. Moreover knockdown of HAS2, CD44 and RHAMM reduced growth and induced apoptosis predominantly in the AGL knockdown bladder cancer cells confirming that loss of AGL drives bladder cancer growth via HAS2/HA/CD44-RHAMM axis. Thus it can be said that reduction in CD44 or RHAMM protein expression with inhibition of HA synthesis is not always a determinant of HA signal inhibition.

It has been reported that CD44 and RHAMM can interact independently with HA to induce certain cellular behavior and in certain cases their interaction have redundant and overlapping functions [22]. We observed something similar in rapid growing bladder cancer cells driven by AGL loss. We observed loss of either CD44 or RHAMM is important for induction of apoptosis in specific AGL low bladder cancer cell line. We observed loss of CD44 induced apoptosis in UMUC3 shAGL cells whereas loss of RHAMM induced apoptosis in T24T and MGHU4 shAGL cells. Apoptosis in these cells was induced by death receptor signaling as implied by increase in DR5 expression. These observations illustrate with AGL knockdown, in some bladder cancer cell lines (UMUC3) inhibition of HA-CD44 interaction, whereas in others (T24T and MGHU4) inhibition of HA-RHAMM interaction is important for induction of death receptor signaling and apoptosis. Change is cellular location of RHAMM with AGL loss in some bladder cancer cell lines (UMUC3) could be a determining factor on how this protein functions. However the reason behind altered RHAMM localization and function with AGL loss in only UMUC3 bladder cell lines is not clear and needs to be investigated. Detailed investigation is required to understand how death receptor signaling and apoptosis is triggered by either CD44 or RHAMM knockdown in rapid growing bladder cancer cells driven by AGL loss. However HA interaction with CD44 and RHAMM has identical effect on bladder cancer cell growth. Anchorage dependent and independent proliferation assay showed that loss of both CD44 and RHAMM inhibited growth of bladder cancer cells driven by AGL loss. This demonstrates that HA interaction with both CD44 and RHAMM gives rise to similar proliferative signaling in the AGL low bladder cancer cells. The mechanism how CD44 and RHAMM drives growth of bladder cancer cells that have lost AGL will be investigated in future.

Inhibitors of CD44 and RHAMM are being tested for cancer treatment with a few inhibitors in clinical trials [7]. However severe side effects due to their effects on normal cells and the immune system have been a major hindrance for drugs targeting CD44 and RHAMM [7]. It is clear for the success of drugs targeted at CD44 and RHAMM it is important to identify the subset of patients whose tumor are highly dependent on CD44 and RHAMM for growth. We propose that bladder cancer patients with low AGL expression are an ideal subset of cancer patients who can be treated with CD44 and RHAMM inhibitors.

## Conclusion

Our previous study and current research have looked in detail into the involvement of HA signaling for aggressive growth of bladder cancer cells driven by AGL loss. We have established that loss of AGL promotes rapid bladder cancer growth via HAS2-HA-CD44/RHAMM pathway. Thus we conclude that inhibition of this pathway at various points can be beneficial for personalized treatment of bladder cancer patients with low AGL expression.

## Additional file

Additional file 1: Table S1. Clinicopathologic characteristics of bladder cancer patient samples in microarray datasets. **Figure S1.** CD44 and RHAMM expression after 4MU treatment in bladder cancer cells +/− AGL. **Figure S2.** CD44 and RHAMM expression after HA treatment in bladder cancer cells +/− AGL. **Figure S3.** CD44 loss and apoptosis in UMUC3 and T24T cells +/− AGL. **Figure S4.** RHAMM loss and apoptosis in UMUC3 and T24T cells +/− AGL. **Figure S5.** Effect of CD44 and RHAMM loss on MGHU4 cell apoptosis +/− AGL. **Figure S6.** Cellular localization of RHAMM in bladder cancer cells +/− AGL. **Figure S7.** HAS2 expression and HA synthesis by bladder cancer cells with AGL after CD44 and RHAMM loss. **Figure S8.** Effect of CD44 and RHAMM loss on MGHU4 cell growth +/− AGL. (DOCX 2675 kb)

## Abbreviations

4MU: 4-Methylumbelliferone
AGL: Amylo-alpha-1-6-glucosidase-4-alpha-glucanotransferase
GSDIII: Glycogen storage disease type III
HA: Hyaluronic acid
HAS2: Hyaluronic acid synthase 2
PYG: Glycogen phosphorylase
RHAMM: Hyaluronan mediated motility receptor
SHMT2: Serine Hydroxymethyltransferase 2
TUNEL: Terminal deoxynucleotidyl transferase (TdT) dUTP Nick-End Labeling
