# Supplementary material for: CD44 and RHAMM are essential for rapid growth of bladder cancer driven by loss of Glycogen Debranching Enzyme (AGL)
Source: BMC Cancer. 2016 Sep 5;16(1):713. doi: 10.1186/s12885-016-2756-5 (PMC5011830; doi:10.1186/s12885-016-2756-5)
Supplement: Additional file 1: Table S1. — Clinicopathologic characteristics of bladder cancer patient samples in microarray datasets. Figure S1. CD44 and RHAMM expression after 4MU treatment in bladder cancer cells +/− AGL. Figure S2. CD44 and RHAMM expression after HA treatment in bladder cancer cells +/− AGL. Figure S3. CD44 loss and apoptosis in UMUC3 and T24T cells +/− AGL. Figure S4. RHAMM loss and apoptosis in UMUC3 and T24T cells +/− AGL. Figure S5. Effect of CD44 and RHAMM loss on MGHU4 cell apoptosis +/− AGL. Figure S6. Cellular localization of RHAMM in bladder cancer cells +/− AGL. Figure S7. HAS2 expression and HA synthesis by bladder cancer cells with AGL after CD44 and RHAMM loss. Figure S8. Effect of CD44 and RHAMM loss on MGHU4 cell growth +/− AGL. (DOCX 2675 kb) [file 12885_2016_2756_MOESM1_ESM.docx]

**CD44 and RHAMM are Essential for Rapid Growth of Bladder Cancer Driven by Loss of Glycogen Debranching Enzyme (AGL)**

Darby Oldenburg^1^, Yuanbin Ru^2^, Benjamin Weinhaus^3^, Steve Cash^1^, Dan Theodorescu^4,5,6*^, Sunny Guin^1#*^

**Supplemental Table 1.** Clinicopathologic characteristics of bladder cancer patient samples in microarray datasets

| **Dataset** | | **Stransky[1]** | **Kim[2]** |
| --- | --- | --- | --- |
| Accession | | E-TABM-147 (ArrayExpress) | GSE13507  (GEO) |
| Platform | | HG-U95Av2 | GPL6102 |
| Sample size | | 41 | 255 |
| Normal samples, n (%) | | 0 (0) | 68 (27) |
| Tumor samples, n (%) | | 41 (100) | 187 (73) |
| Sex, n (%) | Male | 33 (80) | 135 (82) |
|  | Female | 8 (20) | 30 (18) |
| Grade, n (%) | Low | 16 (41) | 105 (64) |
|  | High | 23 (59) | 60 (36) |
| Stage, n (%) | pTa | 12 (29) | 24 (15) |
|  | pT1 | 7 (17) | 80 (48) |
|  | pT2 | 1 (2) | 31 (19) |
|  | pT3 | 10 (24) | 19 (12) |
|  | pT4 | 11 (27) | 11 (7) |
| Node, n (%) | N- | 22 (63) | 149 (91) |
|  | N+ | 13 (37) | 15 (9) |
| Overall survival (months) | Minimal | N.A. | 1.0 |
|  | Maximal |  | 137.0 |
|  | Median |  | 36.6 |
|  | Mean |  | 48.4 |

**Supplemental Figures**

**Supp. Figure 1. CD44 and RHAMM expression after 4MU treatment in bladder cancer cells +/- AGL.** **A.** UMUC3 cells were plated and treated with 4MU at 500 µM concentration. 24 hrs after treatment cells were lysed and Western blot was carried out to detect CD44 and RHAMM expression. **B.** Densitometric analysis of RHAMM and CD44 normalized to Actin and the UMUC3 shCTL siCTL sample (n=3). **C.** T24T cells were plated and treated with 4MU at 500 µM concentration. 24 hrs after treatment cells were lysed and Western blot was carried out to detect CD44 and RHAMM expression. **D.** Densitometric analysis of RHAMM and CD44 normalized to Actin and the T24T shCTL siCTL sample (n=3).

**Supp. Figure 2.** **CD44 and RHAMM expression after HA treatment in bladder cancer cells +/- AGL. A.** UMUC3 and T24T cells +/- AGL were plated and treated with low molecular wt. HA at 50 and 100 μg/ml concentration for 48 hrs. Cells were lysed and Western blot was carried out to detect CD44 and RHAMM expression. **B, C.** Densitometric analysis of CD44 and RHAMM normalized to Actin and the UMUC3/T24T shCTL or shAGL sample without HA treatment (n=3).

**Supp. Figure 3.** **CD44 loss and apoptosis in UMUC3 and T24T cells +/- AGL. A, C.** UMUC3 and T24T shCTL and shAGL cells were plated and 24hrs later transfected with scrambled siRNA (siCTL) or a second siRNA against CD44 (siCD44-2). Details of siRNA are in **Material and Methods**. Cells were lysed 48 hrs after transfection and Western blot was carried out for proteins involved in apoptosis. **B, D.** Densitometric analysis of cleaved apoptotic proteins normalized to total protein and the UMUC3 and T24T shCTL siCTL sample respectively (n=3, *P<0.05).

**Supp. Figure 4.** **RHAMM loss and apoptosis in UMUC3 and T24T cells +/- AGL. A, C.** UMUC3 and T24T shCTL and shAGL cells were plated and 24hrs later transfected with scrambled siRNA (siCTL) or a second siRNA against RHAMM (siRHAMM-2). Details of siRNA are in **Material and Methods**. Cells were lysed 48 hrs after transfection and Western blot was carried out for proteins involved in apoptosis. **B, D.** Densitometric analysis of cleaved apoptotic proteins normalized to total protein and the UMUC3 and T24T shCTL siCTL sample respectively (n=3, *P<0.05).

**Supp. Figure 5. Effect of CD44 and RHAMM loss on MGHU4 cell apoptosis +/- AGL. Ai.** MGHU4 shCTL and shAGL cells were plated and 24hrs later transfected with scrambled siRNA (siCTL) or siGENOME SMARTpool siRNA against CD44(siCD44). Details of siRNA are in **Material and Methods**. Cells were lysed 48 hrs after transfection and Western blot was carried out for proteins involved in apoptosis. **ii.** Densitometric analysis of cleaved Cas 3 normalized to total Cas3 and the MGHU4 shCTL siCTL sample (n=3, *P<0.05). **Bi.** MGHU4 shCTL and shAGL cells were plated and 24hrs later transfected with scrambled siRNA (siCTL) or siGENOME SMARTpool siRNA against RHAMM (siRHAMM). Details of siRNA are in **Material and Methods**. Cells were lysed 48 hrs after transfection and Western blot was carried out for proteins involved in apoptosis. **ii.** Densitometric analysis of cleaved Cas 3 normalized to total Cas 3 and the MGHU4 shCTL siCTL sample (n=3, *P<0.05).

**Supp. Figure 6. Cellular localization of RHAMM in bladder cancer cells +/- AGL. A, B.** UMUC3 and T24T shCTL and shAGL cells were plated in chambered slides. Next day cells were fixed, treated with RHAMM primary antibody and fluorescent secondary antibody and visualized under fluorescent microscope. Detail method under **Material and Methods**. Images were taken at a 40X magnification using Olympus IX71 microscope. Scale 20μm. **C, D.** UMUC3 and T24T shCTL and shAGL cells were plated and 24hrs later transfected with scrambled siRNA (siCTL) or siRNA against RHAMM (siRHAMM). Details of siRNA are in **Material and Methods**. Cells were lysed 48 hrs after transfection and Western blot was carried out for p-P42 and P42 followed by densitometric analysis of p-P42 normalized to total protein and the UMUC3 and T24T shCTL siCTL sample respectively (n=3, *P<0.05).

**Supp. Figure 7.** **HAS2 expression and HA synthesis by bladder cancer cells with AGL knockdown after CD44 or RHAMM loss. A, B.** qRT-PCR demonstrating HAS2 expression in UMUC3 and T24T AGL knockdown (shAGL) cells after CD44 and RHAMM depletion. Cells were plated and 24hrs later transfected with scrambled (siCTL) or directed siRNA against CD44 (siCD44) or RHAMM (siRHAMM). Details of siRNA used are in **Materials and Methods**. Cells were harvested at 72hrs for mRNA followed by qRT-PCR analysis (n=3). **C, D.** HA ELISA to demonstrate HA secreted in the media by UMUC3 and T24T shAGL cells after CD44 or RHAMM loss. Cells were plated and 24hrs later transfected with scrambled (siCTL) or directed siRNA against CD44 (siCD44) or RHAMM (siRHAMM). Details of siRNA used are in **Materials and Methods**. Media was collected 72 hrs after transfection for HA ELISA (n=2). Results are shown as mean±SD, *P<0.05.

**Supp. Figure 8. Effect of CD44 and RHAMM loss and on MGHU4 cell growth +/- AGL. A, B.** 72 hrs after MGHU4 shCTL and shAGL were transfected with siCTL, siCD44 or siRHAMM, they were plated for monolayer growth (n=6) in 96-welled plate (10^3^ cells/well) for 5 days followed by CyQUANT assay. *P<0.05.

**References:**

1. Stransky, N., C. Vallot, F. Reyal, I. Bernard-Pierrot, S.G. de Medina, R. Segraves, Y. de Rycke, P. Elvin, A. Cassidy, C. Spraggon, A. Graham, J. Southgate, B. Asselain, Y. Allory, C.C. Abbou, D.G. Albertson, J.P. Thiery, D.K. Chopin, D. Pinkel, and F. Radvanyi, *Regional copy number-independent deregulation of transcription in cancer.* Nat Genet, 2006. **38**(12): p. 1386-96.

2. Kim, W.J., E.J. Kim, S.K. Kim, Y.J. Kim, Y.S. Ha, P. Jeong, M.J. Kim, S.J. Yun, K.M. Lee, S.K. Moon, S.C. Lee, E.J. Cha, and S.C. Bae, *Predictive value of progression-related gene classifier in primary non-muscle invasive bladder cancer.* Mol Cancer, 2010. **9**: p. 3.
